# Conservation and transmission of seed bacterial endophytes across generations following crossbreeding and repeated inbreeding of rice at different geographic locations

**DOI:** 10.1002/mbo3.662

**Published:** 2018-06-10

**Authors:** Denver I. Walitang, Chan‐Gi Kim, Sunyoung Jeon, Yeongyeong Kang, Tongmin Sa

**Affiliations:** ^1^ Department of Environmental and Biological Chemistry Chungbuk National University Cheongju South Korea; ^2^ Bio‐Evaluation Center KRIBB Cheongju South Korea

**Keywords:** conservation, core microbiota, crossbreeding, recultivation, rice, seed bacterial endophytes, transmission

## Abstract

There are comparatively diverse bacterial communities inside seeds, which are vertically transmitted and conserved, becoming sources of endophytes in the next generation of host plants. We studied how rice seed endophyte composition changed over time following crossbreeding, repeated inbreeding, subsequent human selection and planting of different rice seeds in different ecogeographical locations. Using terminal‐restriction fragment length polymorphism analysis to study bacterial communities, we observed that diversity between the original parents and their offspring may show significant differences in richness, evenness and diversity indices. Heat maps reveal substantial contributions of both or either parent in the shaping of the bacterial seed endophytes of the offspring. Most of the terminal restriction fragments (T‐RFs) of the subsequent progeny could be traced to any or both of its parents while unique T‐RFs of the offspring suggest external sources of colonization particularly when the seeds were cultivated in different locations. Many similar groups of endophytic bacteria persist in the seeds even after recultivation in different locations, indicating resilience to environmental changes and conservation of bacteria across generations. This study suggests that parent plants contributed to the shaping of seed bacterial endophytes of their offspring, although it is also possible that these soil grown rice plants recruit similar populations of endophytes from the soil generation after generation. This study also highlights some bacterial groups belonging to *Herbaspirillum*,* Microbacterium*,* Curtobacterium*,* Stenotrophomonas*,* Xanthomonas and Enterobacter* that may be part of a transmitted and conserved “core microbiota” that are ubiquitous and dominant members of the endophytic communities of the rice seeds.

## 1 INTRODUCTION

Seeds of plants harbor diverse endophytic bacterial communities (Hardoim, Hardoim, Van Overbeek, & Van Elsas, 2012; Johnston‐Monje & Raizada, 2011; Liu, Zuo, Xu, Zou, & Song, 2012; Mundt & Hinkle, 1976). Seed bacterial endophytes are especially interesting because of their intrinsic properties that allow them to colonize plant internal structures including the reproductive parts of the plants and later into the seeds. There are also several modes of colonization by seed bacterial inhabitants and that some of these endophytes are host specific (Escobar‐Rodriguez, Mitter, Barret, Sessitsch, & Compant, 2018). Potentially the most intriguing characteristic of seed bacterial endophytes is their vertical transmission and conservation into the next generation plants (Johnston‐Monje & Raizada, 2011; Truyens, Weyens, Cuypers, & Vangronsveld, 2015). Consecutive seed generations consistently show similar endophytic communities with prominent dominant groups (Sanchez‐Lopez et al., 2018). Additionally, the potential existence of core seed bacterial endophytes was also observed in *Oryza sativa* ssp. indica (Hardoim et al., 2012; Walitang, Kim, Kim, Kang, & Sa, 2018).

The endosphere of plants as a microbiome of bacterial endophytes can also be as dynamic as other systems as it undergoes changes through time and through life cycles. It is constantly affected by inherent factors of the host plant as well as effects of physical environmental changes when plants respond to biotic and abiotic components of its environment. Rice as a host plant affects its endophytic communities through its inner biochemical environment and physiological features unique to the host genotype as the plant undergoes its life changes in completing its life cycle (Okunishi, Sako, Mano, Imamura, & Morisaki, 2005). Rice is also a host to a diverse group of endophytic bacteria with functional characteristics important for endophytic lifestyle (Sessitsch et al., 2012). Furthermore, plants also respond to changes in their environment and consequently affect their associated bacterial endophytes. The plant's substrate is probably the most important of all environmental factors and impacts rice endophytes in a dynamic and ever changing manner (Hardoim et al., 2012).

Studying the factors that change the endophytic bacterial community over time and over host generations is highly interesting—particularly natural crossbreeding, repeated inbreeding and recultivation in different ecogeographic locations. Also, as plants undergo physiological and adaptive modifications in the process of attaining desirable traits such as salinity tolerance and high yield, this may also lead to changes in the bacterial community preferentially retaining endophytes that show competence or establishing a higher degree of host‐microbe symbiosis. In this study, conservation and vertical transmission of seed bacterial endophytes in the original parent lines to the inbred offspring from the original hybrids after natural crossbreeding were investigated together with the effect of recultivation in different ecogeographic locations. The objective is to compare the bacterial community of the original parents to their subsequent offspring and to assess if there are changes in the community structure and diversity between the same rice hosts that were recultivated in different ecogeographic locations. This helps to understand the contributions of parental lineage and recultivation on the diversity and community structure of seed bacterial endophytes allowing conservation of endophytes to the next generation host plants.

## 2 MATERIALS AND METHODS

### 2.1 Seed samples

Rice (*Oryza sativa* L. ssp. indica) seeds of two parental lines and their hybrid offspring that have undergone repeated cycles of inbreeding (creating a recombinant inbred line [RIL]) were included in this study (Table S1). There were two sets of crosses studied. The original crosses include IR29xPokkali and AT401xIR318, which resulted in the RIL offspring FL478 and IC32, respectively. The offspring cultivars were part of a rice breeding process to create a high yielding, salt tolerant rice genotypes. The seeds of the parent lines IR29, Pokkali, AT401 and IR318 were taken from the International Rice Research Institute (IRRI) in the Philippines. The seeds of the offspring lines FL478 and IC32 were taken from the Rural Development Administration (RDA), South Korea. The parental lines Pokkali and AT401 are the putative donors of salinity tolerance genes while IR29 and IR318 are the putative enhancers of yield or are restorer lines. IR29 is a well‐known salt‐sensitive control while Pokkali and FL478 are well‐known salt‐tolerant controls. Aside from the samples from IRRI, IR29 and AT401 seed samples were also acquired from RDA and the effect of recultivation was investigated using these rice cultivars. All seed samples from IRRI and RDA have been maintained as pure RILs making sure that there was no genetic mixing from other rice genotypes. Pokkali has been maintained as a pure wild type cultivar.

### 2.2 Seed surface sterilization and counting of colony forming units

Surface sterilization of rice seeds was done according to Hardoim et al. (2012). Under sterile conditions, decontaminated forceps were used to remove the hulls of rice seeds (1 g). Subsequent surface‐sterilization was done at 30°C for 25 min in an orbital shaker (200 rpm) with a 50 ml solution containing 0.12% sodium hypochlorite (NaClO) and salts (0.1% sodium carbonate, 3% sodium chloride, and 0.15% sodium hydroxide) (Hardoim et al., 2012). Removal of the surface adhered NaClO was achieved by washing with 50 ml 2% sodium thiosulfate (Miché & Balandreau, 2001) repeated twice at 30°C for 10 min under orbital shaking (200 rpm). The seeds were rinsed 5–8 times with sterile distilled water before the seeds were subjected to rehydration for at least 1 hr at room temperature in 100 ml autoclaved demineralized water. The efficiency of sterilization was confirmed by plating 100 μl of the final rinse onto R2A agar plates and incubating them for 7 days at 28°C. Seed samples were discarded when proven to be nonsterile.

Surface sterilized seeds were ground with an autoclaved mortar and pestle. Culturable populations of seed endophytic bacteria were determined by counting the colony forming units (CFU) on R2A (DB—Difco) plates using spread plate technique after serial dilution of the homogenized surface sterilized seed samples (1.0 g). Tenfold serial dilutions were made and 100 µl aliquots were spread onto an R2A agar in three replicates for each dilution. Plates were incubated at 28°C. For bacteria population, counting was done every 24 hr for 6 days.

### 2.3 Total DNA extraction

Total genomic DNA extraction of seeds was done according to Johnston‐Monje and Raizada (2011) with minor modifications. One gram of surface‐sterilized seeds for each genotype was ground in an autoclaved mortar and pestle. One mL of 50 mM Na_2_HPO_4_ buffer per gram of seed dry weight was added. Total genomic DNA was extracted from 0.1 g of extract using DNeasy Plant Mini Kits (Qiagen) following manufacturer's protocol. DNA was also quantified using Nanodrop (Thermo Scientific).

### 2.4 PCR amplification for terminal‐restriction fragment length polymorphism

Seminested PCR conditions for amplification of bacterial DNA were done according to Johnston‐Monje and Raizada (2011). A PCR mastermix was made with the following components: 2.0 μl Standard Taq Buffer, 0.8 μl of 25 mM each of dNTP mix, 0.5 μl of 10 μM 27 F‐Degen primer with sequence AGRRTTYGATYMTGGYTYAG (Frank et al., 2008) (where R = A + G, Y = C + T, M = A + C), 0.5 μl of 10 μM 1492r primer with sequence GGTTACCTTGTTACGACTT (Frank et al., 2008), 0.25 μl of Standard Taq, 20 ng of total DNA, and the final volume was made up to 20 μl with double distilled water. Amplification was performed for 25 cycles in a PTC200 DNA Thermal Cycler (MJ Scientific) using the following program: 96°C for 3 min, 25× (94°C for 30 s, 48°C for 30 s, 72°C for 1 min 30 s), 72°C for 7 min.

The reaction mixture for the seminested PCR consisted of 5.0 μl Standard Taq Buffer, 4.0 μl of 25 mM each of dNTP mix, 2.0 μl of 799f primer, 2.0 μl of 1492r primer, 0.3 μl of Standard Taq, 2.0 μl of 10^−1^ PCR product from the first PCR reaction, and double distilled water up to 50.0 μl total. For the seminested PCR, an antichloroplast primer 799f with sequence AACMGGATTAGATACCCKG (Chelius & Triplett, 2001) (where M = A + C, K = G + T) was labeled with 6FAM, and 1492r primer with sequence GGTTACCTTGTTACGACTT (Frank et al., 2008) were used. The much larger mitochondrial 18S fragments were later removed in silico after amplification and restriction. Amplification of the seminested PCR reaction was performed for 25 cycles in a PTC200 DNA Thermal Cycler (MJ Scientific) using the following program: 95°C for 3 min, 25× (94°C for 20 s, 53°C for 40 s, 72°C for 40 s), 72°C for 7 min.

### 2.5 Restriction enzyme digestion

PCR purification products were digested separately using three restriction enzymes: DdeI, HaeIII and HhaI. For the restriction enzymes, 0.8 μl of 4 U each, 2 μl 10× buffer (buffer C for HaeIII and HhaI), 2 μl of 10× BSA and MilliQ water, adjusted according to the volume of the PCR purification product (1.0 μg/μl) with a total volume of 20 μl. Digestions with HaeIII, HhaI and DdeI enzymes were carried out at 37°C, for 16 hr. All enzymes and reagents were from Promega. Separation and detection of digestion products were carried out by electrophoresis using 2% QA‐agarose TM gel to check for the enzyme digestion. Five microliter of the enzyme digestion products and 6× dye were loaded on the agarose gel.

### 2.6 Sizing

To determine the precise length of the terminal restriction fragments (T‐RFs), 1.5 μl digests were mixed with 9 μl Hi‐Di^™^ formamide (ABI) and 0.6 μl of size standard (500ROX, Bioventures). The samples were denatured at 95°C for 3 min then placed on ice for 5 min. Sizes of restriction fragments were determined on an automated ABI 3130 DNA sequencer (Applied Biosystems). Fluorescent labeled 5′ T‐RFs were detected and analyzed by using Genemapper, ver. 3.7 (Applied Biosystems), with size mapper (500 ROX) designed for sizing DNA fragments in the 50–500 bp range.

### 2.7 Identification of the T‐RFs

To annotate the bacterial taxonomy of the observed T‐RFs, sequences of isolates and clones from previous studies of the same cultivars and related cultivars were submitted to the in silico terminal‐restriction fragment length polymorphism (T‐RFLP) analysis program TRiFLe (Junier, Junier, & Witzel, 2008). Accession numbers used for T‐RF identification are KY862075–KY862113 for clones and KY393309–KY393357 for bacterial isolates.

### 2.8 Analysis

T‐RF peaks identified from individual T‐RFLP profiles were compiled, arranged and adjusted for statistical analysis. To normalize differences in the PCR product quantity and T‐RFLP profile intensity among samples, relative peak area was calculated based on the area of each fluorescent peak divided by the sum of all signals in the corresponding sample (Babendreier, Joller, Romeis, Bigler, & Widmer, 2007). Richness (S) was determined by counting the presence or absence of RF bands in the electrogram. Shannon diversity index (*H*′) was determined using the formula *H*′* *= −∑(*p*_*i*_)(ln *p*_*i*_), while Shannon evenness (*J*′) was calculated as *J*′ = *H*′/ln(*S*), and Simpson index as (1/*D*) = 1/∑*pi* ^2^. In these equations, *p*_*i*_ is for the relative abundance of T‐RFs, ln is for the natural log, *S* is for the number of species and *D* is for Simpson's dominance index, which is inversely proportional to diversity. Comparison of diversity indices between the treatments was done by one‐way ANOVA using SAS (Ver 9.4).

T‐RFLP data set were analyzed by nonmetric multidimensional scaling (NMDS) using Primer V.6 software package. Briefly, each T‐RFLP data set was imported into the Primer V.6 and a similarity matrix was calculated, using Bray Curtis coefficient. The MDS procedure was then used to ordinate the similarity data following 100 random starts. Goodness‐to‐fit or stress was calculated, using Kruskal's stress formula: Stress = √Σ_h*,i*_ *(d*_*hi*_ *−* *ď*_*hi*_ *)* ^*2*^ */Σ*_*h,i*_ *d* ^*2*^_*hi*_), where, *d*_*hi*_ is the ordinated distance between samples *h* and *i*, and *ď* is the distance predicted from the regression. Visualization of the relative abundance using heatmaps was done using matrix2png interface (http://www.chibi.ubc.ca/matrix2png/bin/matrix2png.cgi).

Measurements of similarity between microbial communities as indicated by T‐RFLP were made using Sorensen's similarity index (QS), an indicator of Beta diversity which is useful in comparing microbial communities (Culman, Gauch, Blackwood, & Thies, 2008), using the formula: QS = 2*C*/(S1 + S2) where S1 = total number of species in community 1, S2 = total number of species in community 2, and *C* is the number of species common to the two communities.

## 3 RESULTS

### 3.1 Overall diversity of seed endophytes

A total of six rice cultivars including four parents and their two respective RIL offspring were included in this study. The composition, community structure and diversity of their seed endophytic communities were investigated in relation to seed genotypes and parental lines.

The population density of culturable bacterial endophytic community of rice seeds ranges from 4.50 to 6.65 log CFU/g fresh weight (Table S2) after rehydrating the seeds for 16 hr following surface sterilization. T‐RFLP analyses of the rice seed endophytic community with three enzymes: DdeI, HhaI and HaeIII, revealed considerable complexity with T‐RF richness ranging from 8 to 9, 7 to 9 and 9 to 12, respectively for the IR29‐Pokkali cross and 7–13, 8–9 and 10–12, respectively, for AT401‐IR318 cross (Tables 1 and 2). Diversity indices between IR29, FL478 and Pokkali show statistically significant differences. The same observation was also seen for AT401, IR318 and IC32.

**Table 1:** Diversity indices of bacterial endophytes inhabiting the seeds of IR29, FL478 and Pokkali based on T‐RFLP analysis

Diversity parameter	Rice cultivar	T‐RFLP restriction enzyme profile		
		DdeI	HaeIII	HhaI
Richness	IR29 (♀, SS)	8.33 ± 1.3A	9.33 ± 0.33A	9.66 ± 0.33A
	FL478 (ST)	8.0 ± 0.58A	9.0 ± 0.0B	11.67 ± 1.20A
	Pokkali (♂, ST)	8.0 ± 0.58A	7.33 ± 0.33C	10.00 ± 0.58A
Shannon evenness	IR29 (♀, SS)	0.71 ± 0.02B	0.78 ± 0.01B	0.52 ± 0.02C
	FL478 (ST)	0.83 ± 0.02A	0.65 ± 0.01C	0.88 ± 0.02B
	Pokkali (♂, ST)	0.70 ± 0.02C	0.85 ± 0.02A	0.91 ± 0.01A
Shannon index	IR29 (♀, SS)	1.49 ± 0.07B	1.75 ± 0.03A	1.18 ± 0.03C
	FL478 (ST)	1.73 ± 0.04A	1.43 ± 0.2C	2.17 ± 0.07A
	Pokkali (♂, ST)	1.47 ± 0.10C	1.69 ± 0.03B	2.08 ± 0.03B
Simpson's index	IR29 (♀, SS)	0.67 ± 0.01B	0.75 ± 0.01B	0.49 ± 0.01C
	FL478 (ST)	0.80 ± 0.01A	0.63 ± 0.02C	0.869 ± 0.01A
	Pokkali (♂, ST)	0.67 ± 0.05C	0.78 ± 0.01A	0.867 ± 0.00B

*Note*

Values given are the means of three replicates ±*SE*. Values of the same letter are not statistically significant at *p* < 0.05 (Tukey's test, SAS Version 9.4).

T‐RFLP: terminal‐restriction fragment length polymorphism; SS: salt sensitive; ST: salt tolerant; ♀: female parent; ♂: pollen donor.

**Table 2:** Diversity indices of bacterial endophytes inhabiting AT401, IC32 and IR318 based on T‐RFLP analysis

Diversity parameter	Rice cultivar	T‐RFLP restriction enzyme profile		
		DdeI	HaeIII	HhaI
Richness	AT401 (♀, ST)	7.33 ± 0.33C	9.0 ± 0.0A	11.0 ± 1.0A
	IC32 (ST)	8.33 ± 0.88B	8.0 ± 0.58B	11.0 ± 0.0A
	IR318 (♂, SS)	12.33 ± 0.67A	8.00 ± 0.0B	11.6 ± 0.66A
Shannon evenness	AT401 (♀, ST)	0.6071 ± 0.01C	0.61 ± 0.01A	0.90 ± 0.01B
	IC32 (ST)	0.6107 ± 0.01B	0.50 ± 0.01C	0.92 ± 0.00A
	IR318 (♂, SS)	0.71 ± 0.00A	0.54 ± 0.00B	0.82 ± 0.01C
Shannon index	AT401 (♀, ST)	1.21 ± 0.02C	1.34 ± 0.02A	2.14 ± 0.08A
	IC32 (ST)	1.29 ± 0.07B	1.04 ± 0.05C	2.20 ± 0.01A
	IR318 (♂, SS)	1.77 ± 0.03A	1.12 ± 0.01B	2.02 ± 0.07A
Simpson's index	AT401 (♀, ST)	0.5527 ± 0.0C	0.64 ± 0.01A	0.86 ± 0.01B
	IC32 (ST)	0.5626 ± 0.02B	0.45 ± 0.02C	0.88 ± 0.00A
	IR318 (♂, SS)	0.76 ± 0.01A	0.48 ± 0.00B	0.81 ± 0.02C

*Note*

Values given are the means of three replicates ±*SE*. Values of the same letter are not statistically significant at *p* < 0.05 (Tukey's test, SAS Version 9.4).

T‐RFLP: terminal‐restriction fragment length polymorphism; SS: salt sensitive; ST: salt tolerant; ♀: female parent; ♂: pollen donor.

### 3.2 The endophytic bacterial community structure of rice seeds in the parents and the RIL offspring

**Figure 1:**
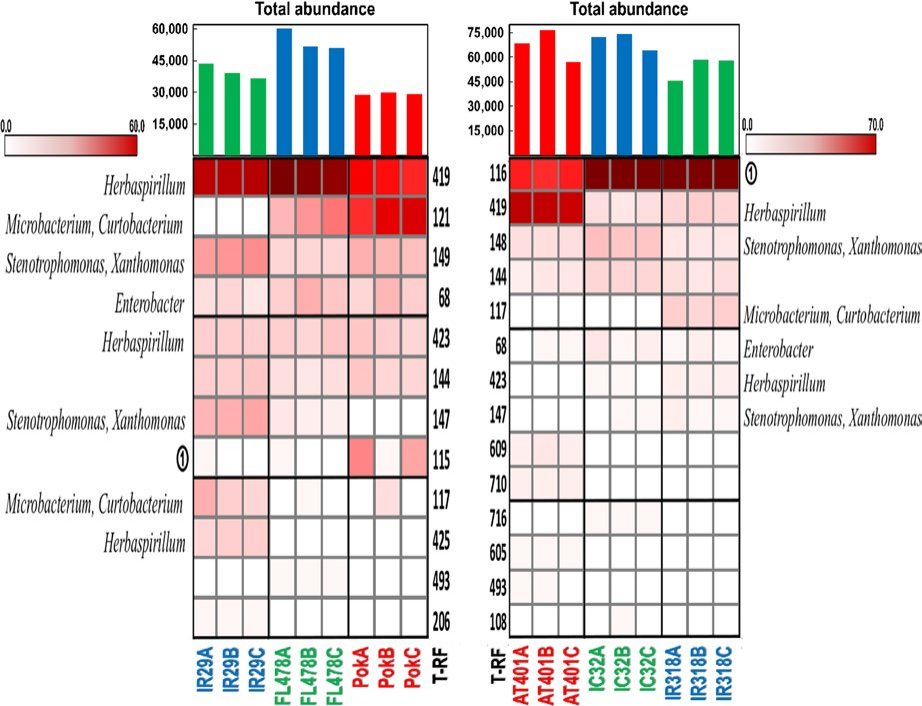
Heat map, relative abundances and total abundance of T‐RFs present in different rice cultivars after digestion with HaeIII. Red labeled cultivars are the salt‐tolerant parents, blue labeled cultivars are the salt‐sensitive/yield enhancer parents and green labeled cultivars are the RIL offspring. T‐RFs are arranged according to decreasing total relative abundance. T‐RF identity: ① *Pantoea*,* Flavobacterium*,* Sphingomonas*,* Delftia*,* Kosakonia*. T‐RFs: terminal restriction fragments; RIL: recombinant inbred line

Several common trends could be recurrently observed in all T‐RFLP profiles of the two separate crosses as a result of three enzyme digestions (Figures 1, S1, and S2). The dominant T‐RFs in any or both of the parents appeared to be the dominant T‐RFs of the progeny as well. There were also common T‐RFs, shared by both parents and their offspring, and genotype‐specific T‐RF's in the different cultivars. The dominant T‐RFs of IR29xPokkali cross belong to *Herbaspirillum* (HaeIII), *Delftia* (DdeI), and *Enterobacter* (HhaI) while *Pantoea*,* Flavobacterium*,* Spingomonas*,* Delftia, Kosakonia* (HaeIII), *Microbacterium* (DdeI), and *Sphingomonas*,* Pseudomonas* and *Enterobacter* (HhaI) appeared to be the dominant bacterial genera in AT401xIR318 cross.

**Figure 2:**
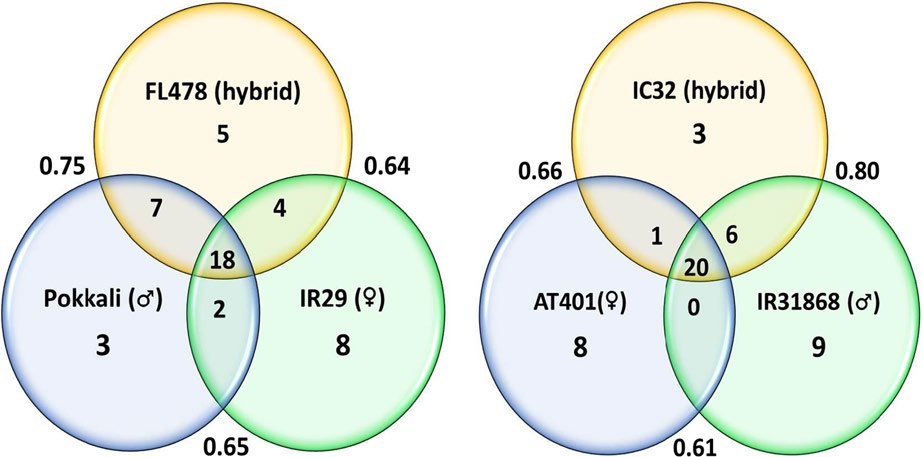
Venn diagrams of the combined T‐RFs present in and among the parent and the hybrid rice cultivars after digestion with DdeI, HhaI and HaeIII restriction enzymes. Outside numbers are Sorensen's similarity index. T‐RFs: terminal restriction fragments

Common T‐RFs were an interesting feature of the endophytic bacterial community observed in both the parents and their offspring. Figure 2 showed that there was an average of 38% and 43% T‐RFs shared between parents and offspring in the IR29xPokkali and IR318xAT401, respectively. The Venn diagrams also show that common T‐RFs shared by the two parents and their offspring are more frequent compared to the cultivar‐specific T‐RFs. These suggest that there are potential “core T‐RFs” or “core microbiota” that are transmitted from both of the parents to their offspring.

Some T‐RFs of the RIL offspring can be traced to only one of its parent. For instance, FL478 shared 7 T‐RFs with its paternal parent, Pokkali, and 4 T‐RFs from its maternal parent, IR29, aside from the common T‐RFs that were shared by all three cultivars. The same pattern can also be observed for the IR318xAT401 cross where IC32 shared 1 T‐RFs to its maternal parent (AT401) and 6 T‐RFs from its paternal parent (IR318).

### 3.3 NMDS analysis

**Figure 3:**
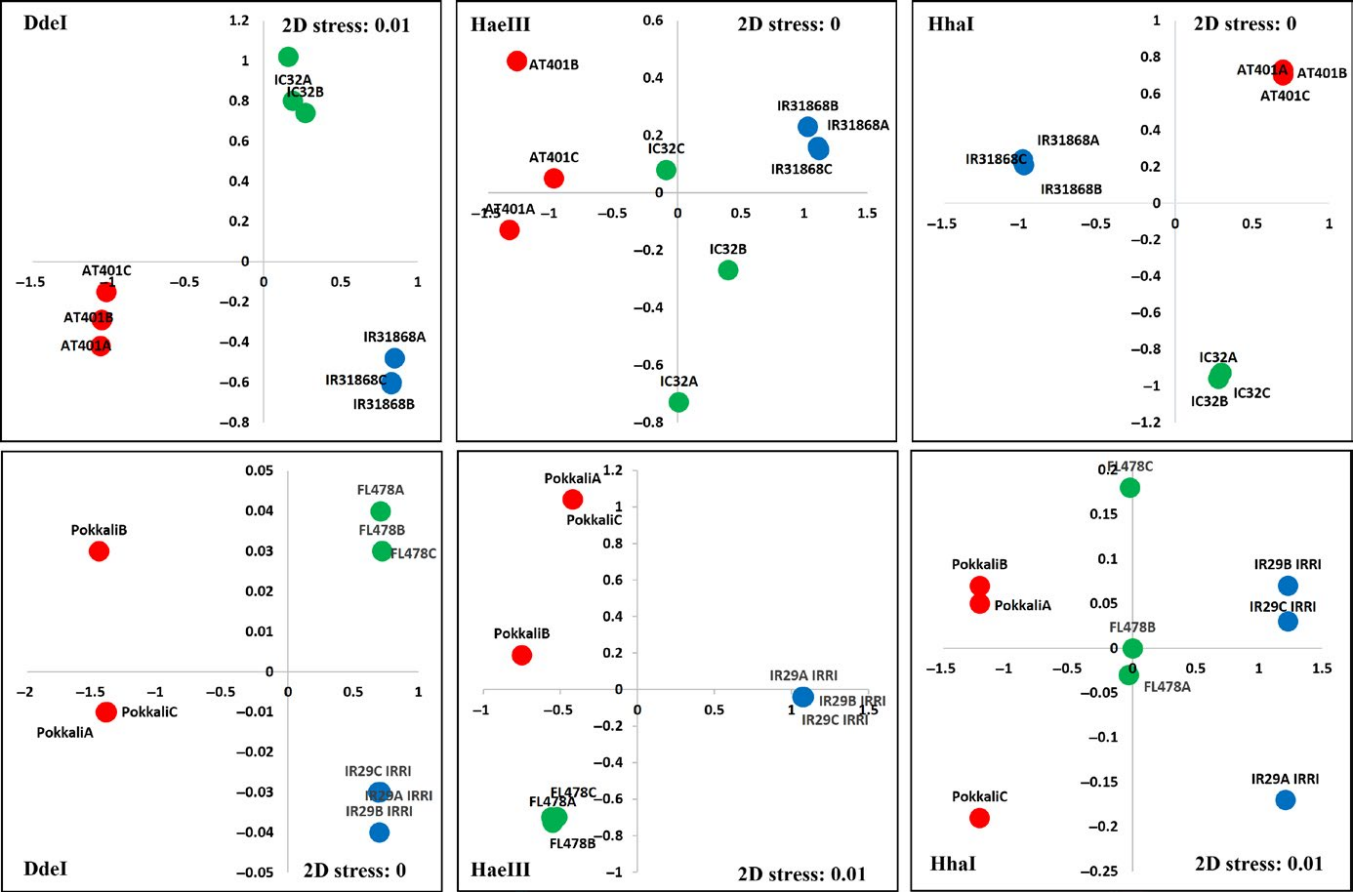
NMDS ordination of IR29xPokkali, AT401xIR318 and their respective RIL offspring FL478 and IC32 based on Bray‐Curtis similarities of the seed endophytic bacterial community T‐RFLP and abundance data after digestion with restriction enzymes DdeI, HaeIII, and HhaI. Red dots are the salt‐tolerant parent cultivars, blue dots are the salt‐sensitive/yield enhancer parents and green dots are the RIL offspring. NMDS: nonmetric multidimensional scaling; RIL: recombinant inbred line; T‐RFLP: terminal‐restriction fragment length polymorphism

NMDS analysis was done to compare overall similarities and differences in endophytic populations based on the T‐RFLP profile. Distances between points in the NMDS ordination in Figure 3 for both crosses show general tight clusters of points belonging to replicates of the same cultivars indicating within group variations is lower compared to between‐group variations. Within cultivars, a tight cluster of points that were well‐separated from other clusters may indicate variation in the bacterial populations from seed to seed which was only observed twice for the green dots in Figure 3. Kruskal's stress values for all the T‐RFLP profiles in the three enzyme digests of both crosses show values equal or below 0.01 indicating good fit and represent a good relationship between points in the matrix.

### 3.4 Diversity of endophytes as a factor of rice cultivation in different ecogeographic location

Comparison of the diversity patterns of the two parental lines (IR29 and AT401) show differences in the way the structure of their seed bacterial endophytes change due to recultivation in different ecogeographic locations. IR29, a commonly used salt‐sensitive control, did not show any significant change in its richness. Richness ranges from 7 to 18, 8 to 9 and 10 to 11 in DdeI, HaeIII, and HhaI T‐RF profiles, respectively (Table 3). Evenness seems to have increased when IR29 was recultivated in RDA which caused diversity indices, Shannon Index and Simpson's Index to increase as well. AT401 showed an opposite trend compared to IR29. Though richness did not change, diversity indices decreased in AT401 samples maintained in RDA, South Korea.

**Table 3:** Diversity indices of bacterial endophytes inhabiting the seeds of IR29 and AT401 based on T‐RFLP analysis

Diversity parameter	Rice cultivar	T‐RFLP restriction enzyme profile		
		DdeI	HaeIII	HhaI
Richness	IR29‐IRRI	8.7 ± 1.20B	9.33 ± 0.33A	10.67 ± 0.33A
	IR29‐RDA	15.3 ± 3.18A	8.67 ± 0.33A	11.33 ± 0.33A
	AT401‐IRRI	7.7 ± 0.33A	9.0 ± 0.0A	11.0 ± 1.0A
	AT401‐RDA	7.0 ± 0.58A	7.67 ± 0.33B	9.33 ± 0.33A
Shannon evenness	IR29‐IRRI	0.71 ± 0.02A	0.78 ± 0.01B	0.74 ± 0.02B
	IR29‐RDA	0.78 ± 0.01B	0.89 ± 0.01A	0.89 ± 0.02A
	AT401‐IRRI	0.61 ± 0.01B	0.62 ± 0.02A	0.90 ± 0.01A
	AT401‐RDA	0.66 ± 0.01A	0.62 ± 0.0A	0.72 ± 0.02B
Shannon index	IR29‐IRRI	1.51 ± 0.06B	1.76 ± 0.03B	1.74 ± 0.03B
	IR29‐RDA	2.08 ± 0.2A	1.93 ± 0.03A	2.16 ± 0.02A
	AT401‐IRRI	1.23 ± 0.01A	1.37 ± 0.05A	2.14 ± 0.08A
	AT401‐RDA	1.28 ± 0.08A	1.26 ± 0.02B	1.61 ± 0.07B
Simpson's index	IR29‐IRRI	0.68 ± 0.01B	0.75 ± 0.01B	0.73 ± 0.01B
	IR29‐RDA	0.83 ± 0.04A	0.84 ± 0.00A	0.87 ± 0.01A
	AT401‐IRRI	0.56 ± 0.01B	0.65 ± 0.02A	0.86 ± 0.01A
	AT401‐RDA	0.65 ± 0.04A	0.61 ± 0.01B	0.71 ± 0.02B

*Note*

Values given are the means of three replicates ±*SE*. Values of the same letter are not statistically significant at *p *< 0.05 (Tukey's test, SAS Version 9.4).

T‐RFLP: terminal‐restriction fragment length polymorphism; IRRI: International Rice Research Institute; RDA: Rural Development Administration.

### 3.5 Comparison of the T‐RFLP profiles between cultivars cultivated in IRRI and RDA

**Figure 4:**
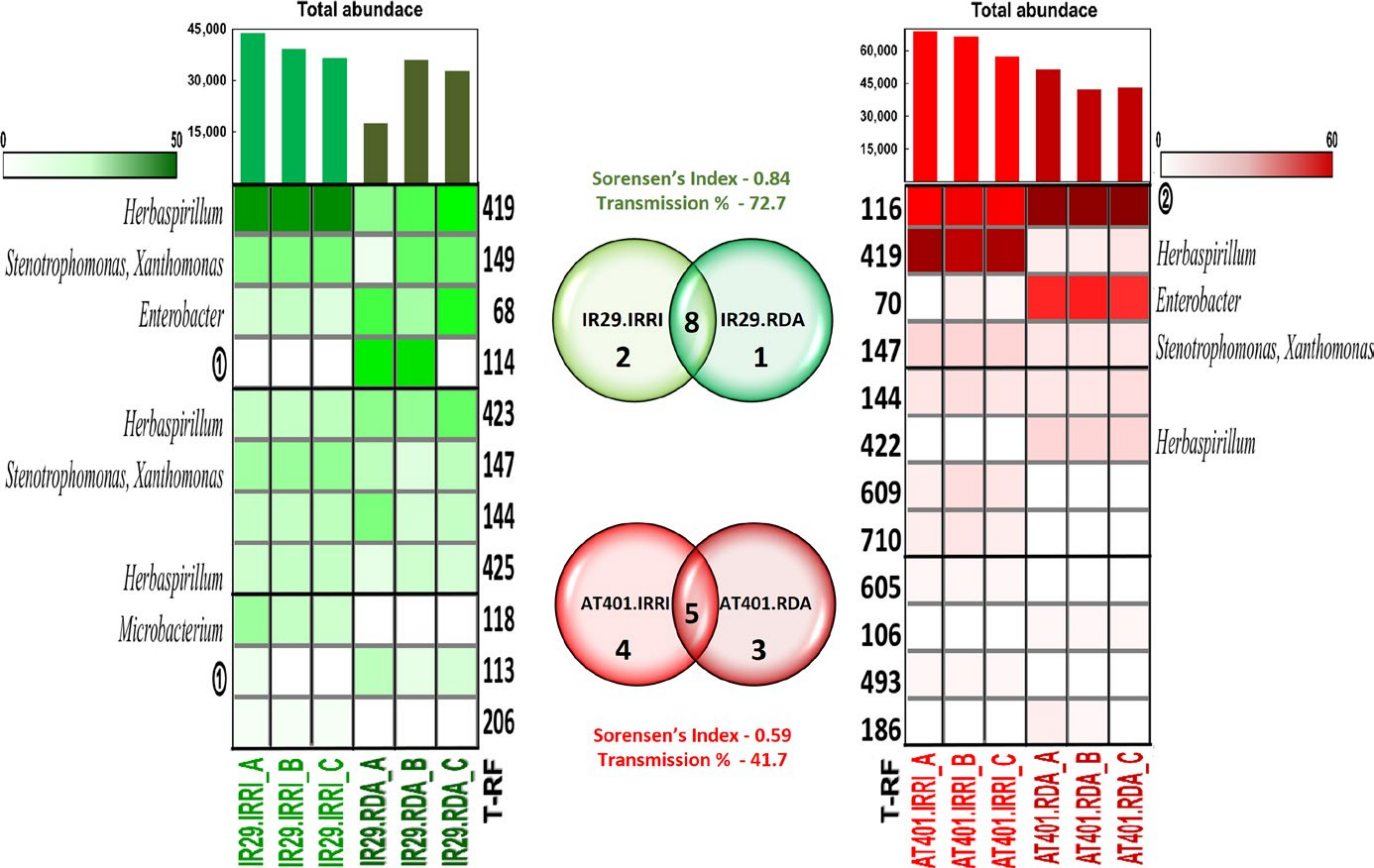
Heat map, relative abundances, total abundance and Venn diagrams of T‐RFs present in rice cultivars grown in IRRI, Philippines and RDA, Korea after digestion with HaeIII. The green heatmap represents the salt‐sentive cultivar, IR29, and the red heatmap represents the salt‐tolerant cultivar, AT401. T‐RFs are arranged according to decreasing total relative abundance. T‐RF identity: ① *Pantoea*,* Flavobacterium*,* Delftia*,* Sphingomonas*,* Pseudomonas*,* Kosakonia*; ② *Pantoea*,* Flavobacterium*,* Microbacterium*,* Sphingomonas*,* Pseudomonas*,* Kosakonia*. T‐RFs: terminal restriction fragments; RDA: Rural Development Administration; IRRI: International Rice Research Institute

The comparison of endophytic bacterial community profiles of each cultivar shows general patterns in the community structure, abundance and similarities after cultivation in different ecogeographic locations (Figures 4, S3, and S4). There is a very high similarity in endophytic composition between seeds even after recultivation in different locations, with the dominant bacterial T‐RFs of the cultivar cultivated in one location usually remaining as the dominant bacterial T‐RFs even after cultivation at another location.

The empirical similarity between the samples cultivated in different ecogeographic locations shows high resemblance when Sorensen's coefficient is taken into consideration (Table 4; Figure 4). IR29, from IRRI and RDA, has Sorensen's index of 0.67, 0.88 and 0.84 in DdeI, HhaI, and HaeIII T‐RFLP profiles, respectively. AT401 shows a coefficient of 0.89, 0.73 and 0.59 in DdeI, HhaI, and HaeIII profiles, respectively. Looking into the details of the endophytic communities, there are many of the same sizes and intensities of T‐RFs found between the same cultivars even after recultivation in different locations. On the other hand, Sorensen's coefficient between IR29 and AT401 cultivated in the same or different ecogeography ranges from 0.43 to 0.69 showing that there is variation from seed to seed as well as from genotype to genotype. Sorensen's coefficient of the same genotype of rice cultivated in different ecogeoraphic location is consistently higher compared to different genotypes cultivated in the same or different lands.

**Table 4:** Beta diversity comparison using Sorensen's similarity coefficient between bacterial endophytic community inhabiting the seed endosphere of rice cultivar IR29 and AT401 cultivated in IRRI, Philippines and RDA, South Korea

Seed community being compared	DdeI	HaeIII	HhaI
IR29.IRRI versus IR29.RDA	0.67	0.84	0.88
IR29.IRRI versus AT401.IRRI	0.6	0.42	0.5
IR29.IRRI versus AT401.RDA	0.6	0.44	0.55
IR29.RDA versus AT401.IRRI	0.43	0.44	0.66
IR29.RDA versus AT401.RDA	0.43	0.55	0.69
AT401.IRRI versus AT401.RDA	0.89	0.59	0.73

*Note*

IRRI: International Rice Research Institute; RDA: Rural Development Administration.

That being said, when seeds were produced at different locations, there were some new endophytes most probably originating from the soil and the new environment to which they grew. This colonization was observed as new T‐RF signals only found in specific samples and not shared between samples of the same cultivar. These new T‐RFs were observed in all the T‐RFLP profiles such as T‐RF 191 and 356 in HhaI for IR29 (Figure S4).

## 4 DISCUSSION

In an earlier study (Walitang et al., 2018) which examined the bacterial endophytes of rice cultivars belonging to the *Oryza sativa* L. ssp. indica, we observed that there are core microbial communities found in all the indica subspecies and that the endophytic bacterial communities and diversity are influenced mainly by the host genotype, physiological adaptation to salinity stress and partly by phylogenetic relatedness. These results suggest that the offspring as products of crossbreeding experiments could also inherit different microbiomes from their parents. In this study, we compared the diversity and community structure of two RIL progenies and their respective parental lines. Our results show a striking similarity between bacterial population in seeds of the parental lines and the bacterial communities of their offspring (Figures 1, S1, S2, and 3) as most of the endophytes of the offspring could be traced to any or both of the parents (Figure 2). The similarity between T‐RFLP profiles could suggest that bacterial populations in the seed are dominant parts of the mature plant microbiome, and that parent plants transmit the majority of their bacterial endophytes into the next generation of seeds.

This study also investigated the community structure and diversity of populations of endophytic bacteria in rice (*Oryza sativa* L. spp. indica) seeds in two pure inbred lines, IR29 and AT401, as they are self‐pollinated and recultivated in different ecogeographic locations, IRRI in Philippines and RDA in South Korea. T‐RFLP analysis was used to gain an overview of the changes, shifts and resilience to change of the endophytic bacterial communities. The endophytic bacterial community of these rice seeds showed stability in the structure and diversity (Table 3) after being recultivated in different ecogeographic locations, with particular endophytic groups reproducibly seen to colonize the seed endosphere (Figures 4, S3, and S4). As the rice endophytic community is consistently shaped by vertical transmission from seed to seed and colonization from outside sources particularly the soil, it appears that seed vertical transmission could play a more important role because seed populations vary little from generation to generation.

### 4.1 Parents could directly or indirectly shape the endophytic community of their offspring

Most of the endophytic bacteria of each offspring seed could be traced to either or both of the parents. There is a high degree of similarity as indicated by Sorensen's indices between each parent and their offspring. Ordination of the bacterial communities in NMDS shows that the offspring is usually situated in the middle or closer to one parent but never situated extremely far from any parent signifying that each parent contributed to the shaping of endophytic communities of their respective offspring. All of these imply direct or indirect mechanisms that allow similar groups of bacterial community to occur between the parents and their offspring after natural crossbreeding events and repeated cycles of inbreeding, recultivation, and selection in order to maintain pure breeding or inbred lines.

Vertical transmission of bacterial endophytes from the parent plant to seed offspring is one way that bacteria could travel through generations of rice via seeds (Mastretta et al., 2010). Bacteria colonizing reproductive structures of plants, then the seeds, are also of special interest to biotechnology because they could be vertically transmitted (Compant, Clément, & Sessitsch, 2010). Hardoim et al. (2012) showed that 45% of the bacterial community from the first generation rice seed was found in the second generation seeds as well. They noted that rice seed endophytes initially colonized the roots then rapidly migrated to the shoots. Similarly, transmission of endophytes in maize seeds following migration and recultivation also resulted in an average of 13%–22% with some cultivars having a vertical transmission as high as 44% between two immediate generations of maize seeds cultivars (Johnston‐Monje & Raizada, 2011). Both studies showed that seed endophytes can be transmitted from generation to generation through the seeds.

Direct vertical transmission is more likely through the maternal parent than through pollen as rice florets are self‐pollinated (Matsui & Kagata, 2003). This detail of seed endophyte transmission is also true for fungal endophytes of grasses (Saikkonen, Wali, Helander, & Faeth, 2004). Male parents may also be able to transmit endophytes to their progeny as there were several studies proving presence of bacteria colonizing the pollen grain of plants. For example, Furnkranz et al. (2012) showed that Gammaproteobacteria, Alphaproteobacteria and Firmicutes, dominant endophytic groups, are found to colonize the surface of pumpkin pollen. Heydenreich et al. (2011) also noted the dominance of Gram‐positive bacteria colonizing the surfaces of timothy grass pollen grains. Pollen may be able to transmit these microbes to the female egg cell where upon fertilization, they could become incorporated into the carposphere and spermosphere of plants.

Contrary to vertical transmission of endophytes through seeds, similar plant genotypes may simply select similar populations of putative endophytes from the surrounding environment. For example, one of the most important factors that start the plant–microbe interaction in the rhizosphere region is colonization by putative endophytes. Competition for root niches starts with the recognition of specific compounds in the root exudates by the bacteria (Compant, Duffy, Nowak, Clément, & Barka, 2005; Compant et al., 2010). Plants may exert selective pressure on the type of bacterial communities they may associate with via their root exudates. In turn, competent bacterial communities will optimize their metabolism toward a physiological state that endows maximal nutrient acquisition, competition and growth in response to exudates secreted from the roots (Hardoim, van Overbeek, & van Elsas, 2008). Parents may indirectly shape their offspring's endophytic communities through production of related exudates. Consequently, similar physiology directly attributed to the inherited genes of the parents could indirectly affect the endophytic microbiota of the offspring. In a comparative study of rice root and seed endophytes, the indica subspecies tend to contain similar groups of bacteria and their composition and diversity is largely influenced by plant genotype across rice cultivars (Hardoim et al., 2011; Walitang et al., 2018). Also, Ding, Palmer, and Melcher (2013) observed that there are significant differences in endophytic communities between different species but hosts from similar phylogenetic lines were more similar than distantly related hosts. On a larger scale, one study showed that seed endophytes reflect maize phylogenetic relationships and that core endophytes are transmitted and conserved beyond effects of evolution, human use and ecology (Johnston‐Monje & Raizada, 2011). There are also other potential sources of colonization by seed bacterial inhabitants (Escobar‐Rodriguez et al., 2018) although for maize and rice, these appear to be less important than seed transmission.

### 4.2 The dominant bacterial communities of the parents could also become the dominant bacterial endophytes of their offspring

In general, the abundant bacterial groups of the offspring were also the abundant endophytes of its respective parents in all the T‐RFLP profiles produced using different enzyme digestion. In most cases too, genotype‐specific T‐RFs are usually the less abundant T‐RFs. The dominance of similar groups of bacteria in parent and offspring was also observed in cloned bacterial sequences of phylogenetically related *Zea* host plants (Liu et al., 2012). They attributed the dominance of similar types of endophytes to the relatedness in terms of genetic similarities as well as physiological similarities of the parents and their respective offspring.

Though host genotype and physiology greatly affect the selection of seed endophytes, the dominance of similar groups of bacteria found in the parents and their offspring indicates a more stable coevolution in terms of host–microbe interaction. Though there are debates on the functional roles of dominant species and diverse communities, dominant endophytes could potentially provide functional importance to the growth, development, and health of their host plant as many of the isolated endophytes show plant growth promoting abilities (Hardoim et al., 2008; Johnston‐Monje & Raizada, 2011; Walitang et al., 2017). We could even speculate in this study as have others (Sasaki & Laurenroth, 2011) that because the dominant species are commonly transmitted and conserved by the host plants, they regulate temporal stability in the endophyte communities. There is also a primary focus on dominant species as biotic controllers of ecosystems processes (Loreau et al., 2011), though in this case, these processes involve plant host–endophyte interactions. The endosphere competence of bacterial endophytes should also be taken into consideration. Bacterial colonization and survival in the host plant is not a random process but also constitutes a form of selection as endophytes capable of responding to host and environmental changes could colonize the host (Hallmann, Quadt‐Hallmann, Mahaffee, & Kloepper, 1997; Hardoim et al., 2008; Walitang et al., 2017). Only specialized and competent endophytes could colonize and survive in the reproductive organs of plants (Compant et al., 2010; Hardoim et al., 2008; Okunishi et al., 2005). Bacterial isolates from the same cultivars and related cultivars also have putative endophytic adaptations that may allow them to survive and colonize the plant endosphere particularly the seeds (Walitang et al., 2017). It is not surprising if the most adapted bacteria could also be the more dominant endophytes in the microbial community of the rice seeds.

### 4.3 There are dynamic changes that occur in the seed microbial community

Like any microbial community, endophytic populations are also influenced by a combination of factors such as bacterial inocula (Andreote, Rocha, Araújo, Azevedo, & Van Overbeek, 2010; Conn & Franco, 2004), pathogens (Sessitsch, Reiter, Pfeifer, & Wilhelm, 2002), location and plant species (Ding et al., 2013) and soil (Hardoim et al., 2012; Johnston‐Monje, Mousa, Lazarovits, & Raizada, 2014). As the seeds are transported and recultivated in different ecogeographic locations, changes may occur in the endophytic community. Though soil offers potentially new endophyte colonizers, colonization still depends on the host plant and the indigenous plant‐associated microbial community (Andreote et al., 2010). Nonnative bacteria colonizing the plant endosphere have to be competent (Hardoim et al., 2008). They also need to survive the defences of the plant immune reaction and modulate succeeding responses (Balmer, Paster, Gamir, Flors, & Mauch‐Mani, 2015; Pieterse et al., 2014). After successful colonization, new endophytic colonizers have to maintain interaction with its host plant and the indigenous endophytes (Brader et al., 2017). It was observed in this study that diversity in terms of richness, evenness and relative abundance may fluctuate, but the common and usually the dominant T‐RFs are generally the same endophytic groups in the seeds even after recultivation. These suggest that bacterial communities of the seed are formed primarily through vertical transmission from the parent plant, even though some endophytes can originate from other external sources.

### 4.4 There are core endophytic populations that are resilient to changes allowing transmission and conservation to the next generation through the seeds

The seed endophytic community of the two separate samples, IR29 and AT401, cultivated in different ecogeographic locations each showed striking similarities with their respective endophytic populations. The IR29 samples from IRRI and RDA showed high similarity with the same T‐RFs. This is also true for samples of AT401. The high similarity indicates that either the seeds of the same genotypes were able to faithfully transmit the majority of their endophytes to the next generation of seed or that each generation of rice seed is able to take up similar populations of endophytes from the soil. Many seed borne endophytes were maintained in terms of presence and abundance even if the seeds were cultivated in different ecogeographic locations. In addition, there is a relative similarity between the different indica cultivars cultivated in the same or in different locations. It has already been observed that there are common seed endophytes in *Oryza sativa* ssp. *indica* (Walitang et al., 2018). The results of this study support the possibility that ancestral parental lines of indica rice cultivars continuously transmit their bacterial endophytes through the seeds allowing conservation and dispersal to succeeding generations of modern rice cultivars.

There are many common T‐RFs found between the parents and their respective offspring. These T‐RFs belong to *Herbaspirillum*,* Microbacterium*,* Curtobacterium*,* Stenotrophomonas*,* Enterobacter*,* Delftia*,* Sphingomonas*,* Pseudomonas*,* Xanthomonas*,* Pantoea*,* Flavobacterium, Pseudomonas*, and *Kosakonia*. Bacterial groups belonging to *Curtobacterium*,* Flavobacterium*,* Enterobacter*,* Xanthomonas*,* Herbaspirillum*,* Microbacterium*, and *Stenotrophomonas* have been found to be core bacterial groups of *Oryza sativa* spp. indica either through T‐RFLP analysis or through sequencing of isolates and clones (Walitang et al., 2017, 2018). In the present study, these bacterial groups have been found to be common in both parents and offspring and generally do not disappear even during repeated inbreeding and recultivation in different ecogeographic locations. Hardoim et al. (2012) found that *Stenotrophomonas maltophilia*, the closest bacterium identified in the previous study through clones (Walitang et al., 2018) was a core endophyte of *Oryza sativa* cv. APO. *Pantoea ananatis* (also the same bacterial identity in the previous study), *Pseudomonas syringae* and *Brevundimonas* sp. were also found to be potential core endophytes of rice and are mainly prominent in the leaves (Ferrando, Mañay, & Scavino, 2012). *Clostridium* and *Paenibacillus* were core endophytes of Zea while *Enterobacter*,* Methylobacteria*,* Pantoea*, and *Pseudomonas* were also found to be widespread among Zea cultivars (Johnston‐Monje & Raizada, 2011). Gammaproteobacteria comprise a large number of bacterial endophytes represented mainly by a few genera particularly *Pseudomonas*,* Enterobacter*,* Pantoea*,* Stenotrophomonas*,* Acinetobacter*, and *Serratia* (Hardoim et al., 2011, Hardoim et al., 2015). These suggest highly evolved interaction of core microbiota with their host plants and represented by some bacterial genera. Some of these bacterial groups have also been isolated in many indica subspecies and each bacterial group may even be represented by a single bacterial species. Furthermore, the bacterial isolates belonging from these groups were also proven to promote growth and germination during the early stages of seed development potentially attributed to their multiple plant growth promoting characteristics (Walitang et al., 2017). These groups were also observed as usual endophytes of rice (Elbeltagy et al., 2001; Sun et al., 2008; Ferrando et al., 2012; Kaga et al., 2009; Hardoim et al., 2012; Sessitsch et al., 2012) isolated from different endosphere regions. These suggest their high association with the rice host and the occurrence of core endophytes of *Oryza sativa* spp. indica. Under salt stress conditions, for which the cultivars are screened for tolerance, certain groups including *Flavobacterium*,* Pantoea*,* Curtobacterium*,* Microbacterium*,* Kosakonia*, and *Enterobacter* become more dominant in terms of abundance. This indicates their potential functional role in enhancing tolerance of their rice host (Walitang et al., 2018). Transmission and conservation of these groups into succeeding generations of rice hosts through the seeds even after crossbreeding and recultivation also points a coevolutionary existence between the rice host and its bacterial endophytes.

Direct vertical transmission of seed endophytes to the immediate next generation seeds have already been proven by Hardoim et al. (2012), Johnston‐Monje and Raizada (2011) and Sanchez‐Lopez et al. (2018). Highly interesting results from their study also showed that the mature second generation seeds had bacterial endophytic populations that showed a strikingly similar pattern of the community as the first generation seeds. They have also shown that seed endophytes can colonize the rhizosphere, vascular tissues and the plant endosphere where they systematically migrate to other plant organs and eventually end up in the seeds. Johnston‐Monje et al. (2014) later showed that the largest fraction of 16S signals in a maize genotype grown in different soils was due to transmission of bacteria from seed to seed.

## 5 CONCLUSIONS

The findings of the study show that parental lines influence the community structure and diversity of the seed bacterial endophytes of their offspring rice plants. Shared bacterial endophytes are also transmitted to a higher degree than genotype‐specific T‐RFs and these common T‐RFs are generally the more dominant bacterial groups of the parents and their offspring. Recultivation of rice in different ecogeographic location may cause shifts and fluctuations in the community structure and diversity of rice seed endophytes in subsequent generations of seed, but the majority of the seed endophytes, especially the dominant groups, are maintained. These shared and dominant groups include core seed bacterial endophytes belonging to *Herbaspirillum*,* Microbacterium*,* Curtobacterium*,* Stenotrophomonas*,* Xanthomonas*, and *Enterobacter*. Other dominant groups include *Delftia*,* Sphingomonas*,* Pseudomonas*,* Flavobacterium*, and *Pantoea*. Results show that there is resilience, transmission and conservation of rice seed bacterial endophytes, especially core microbiota, beyond the process of crossbreeding, human selection, repeated inbreeding and recultivation in different ecogeographic locations. Plant–endophyte interaction should be deeply investigated to understand the dynamic contributions of seed endophytes and core microbiota to their plant host.

## AUTHOR CONTRIBUTION

D.W. and T.S. conceived and designed the experiments; D.W. performed the experiment and analyzed the data; D.W. wrote the manuscript; T.S. critical revision of the manuscript; C.K. multivariate analysis; D.W., C.K., S.J., Y.K., and T.S. manuscript preparation and editing. All authors read and approved the final manuscript.

## CONFLICT OF INTEREST

The authors declare that they have no competing interests.

## AVAILABILITY OF DATA AND MATERIALS

All data generated or analyzed during this study are included in this article (and its supplementary information files) or are available from the corresponding author on reasonable request.
