# Supplementary figures and images for: Conservation and transmission of seed bacterial endophytes across generations following crossbreeding and repeated inbreeding of rice at different geographic locations
Source: Microbiologyopen. 2018 Jun 10;8(3):e00662. doi: 10.1002/mbo3.662 (PMC6436425; doi:10.1002/mbo3.662)

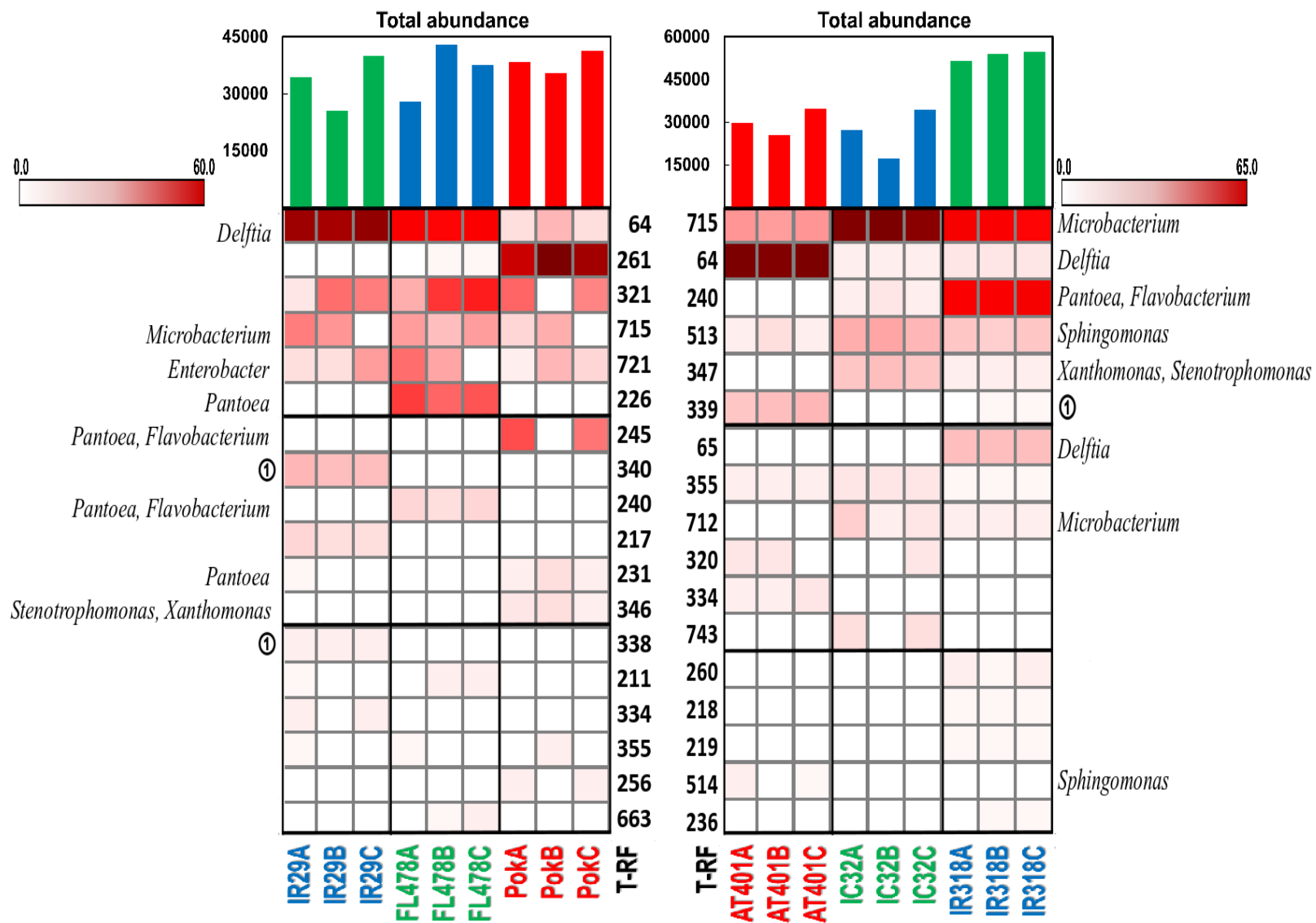

Supplement: Supplementary file 1 [file MBO3-8-e00662-s001.pdf]

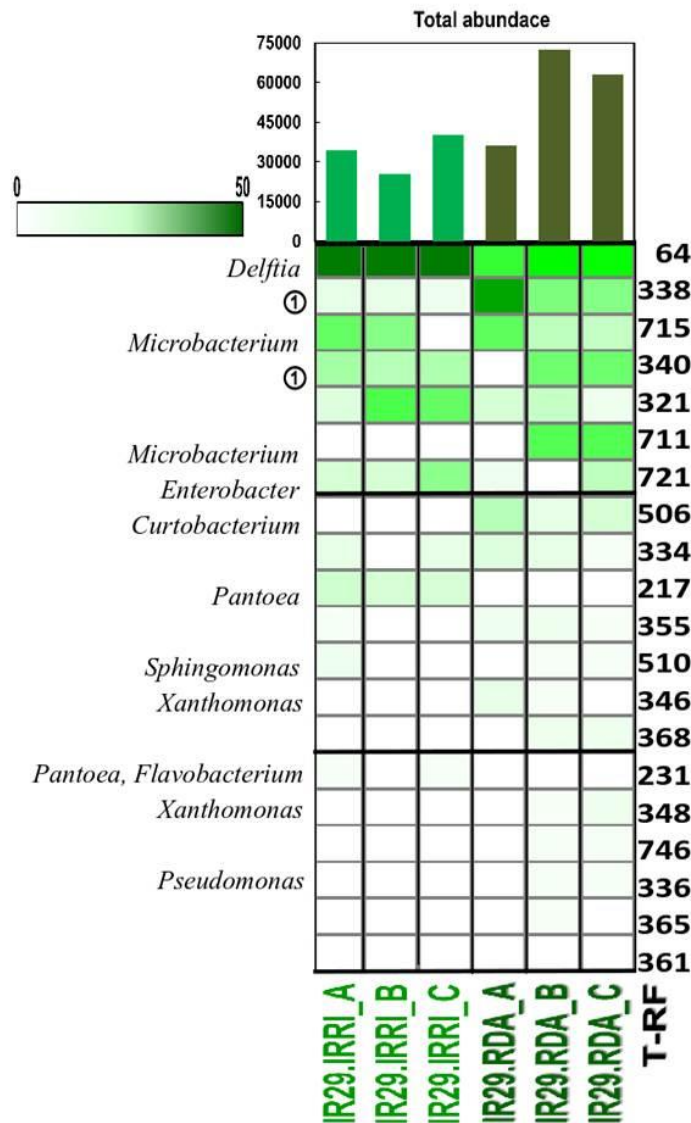

Sorensen's Index - 0.67  
Transmission % - 50

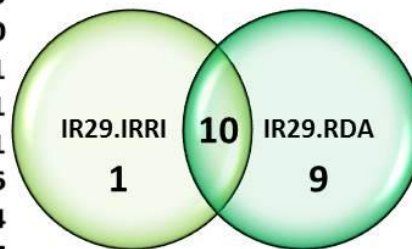

Sorensen's Index - 0.89  
Transmission % - 80.0

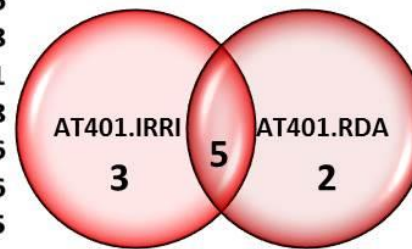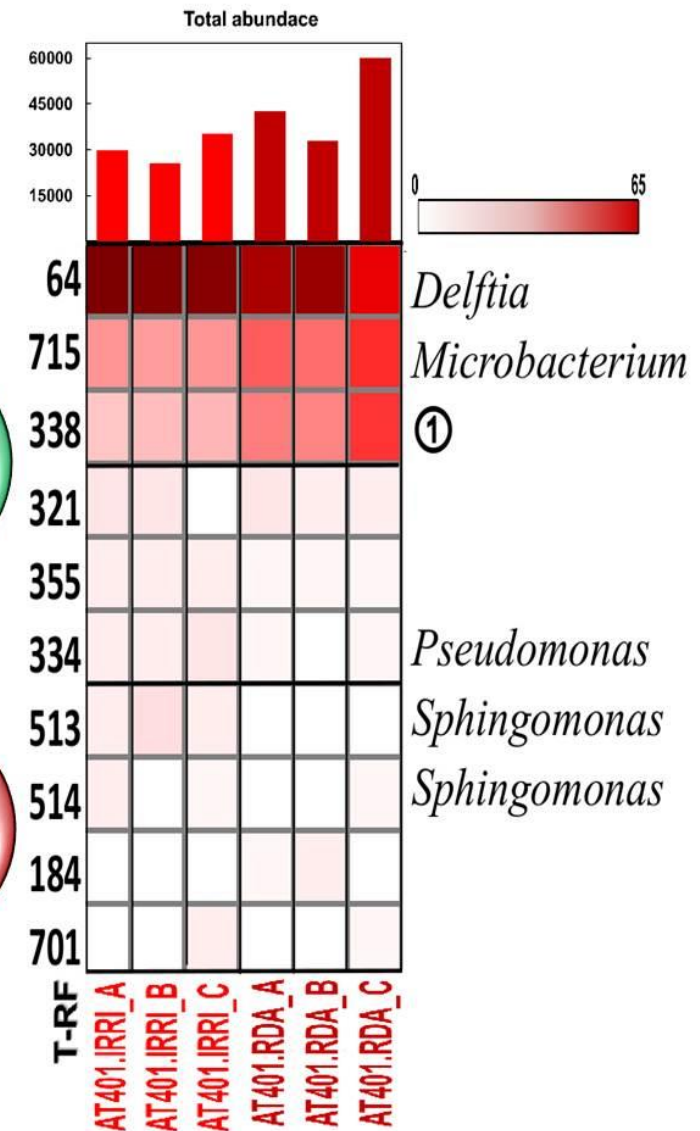

Supplement: Supplementary file 3 [file MBO3-8-e00662-s003.pdf]

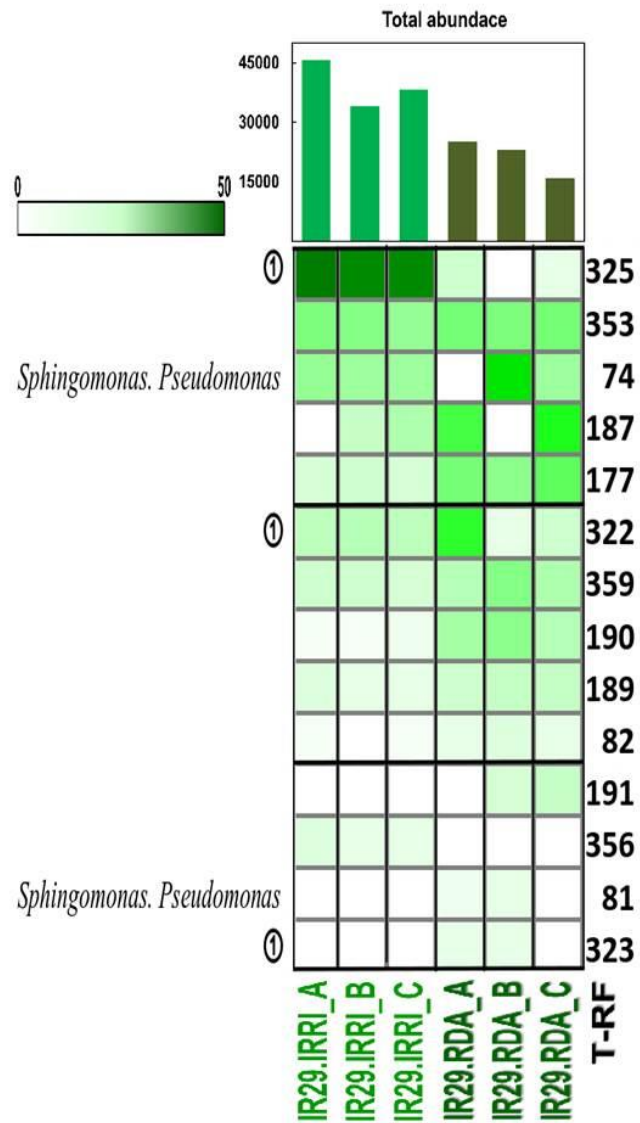

Sorensen's Index - 0.88  
Transmission % - 78.6

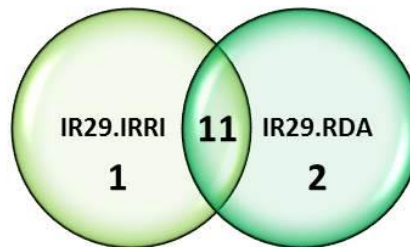

Sorensen's Index - 0.73  
Transmission % - 57.1

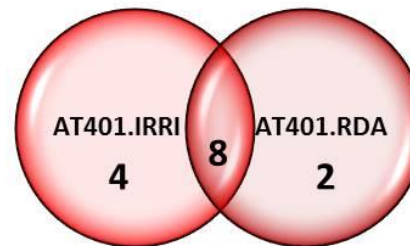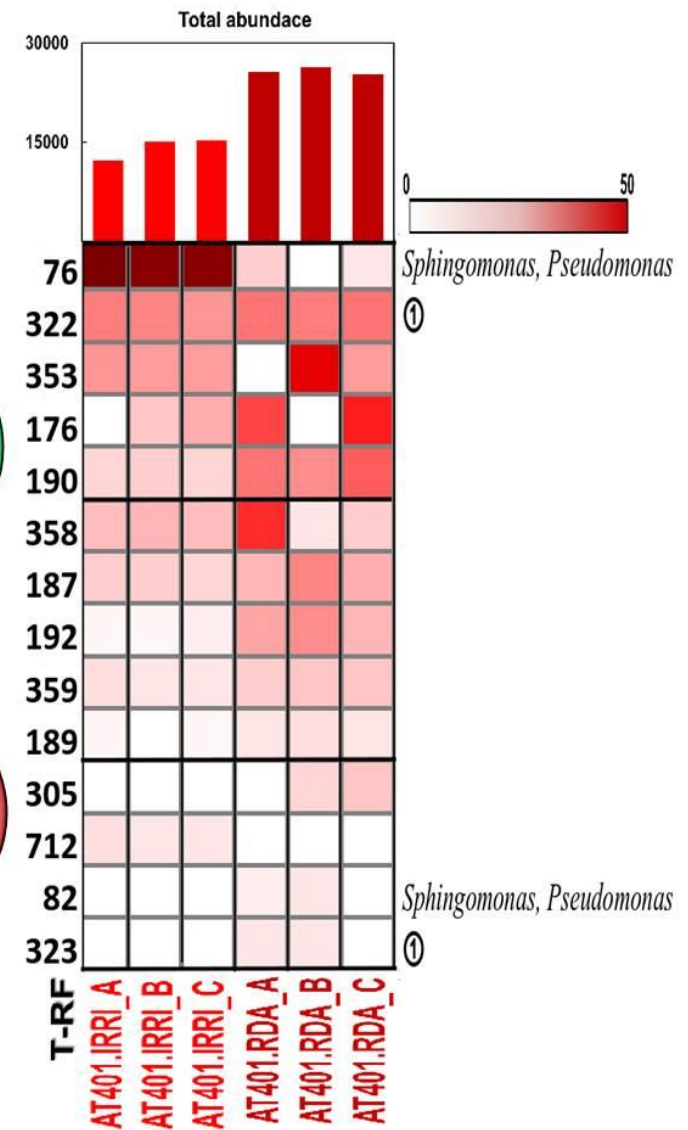

Supplement: Supplementary file 4 [file MBO3-8-e00662-s004.pdf]
