# Supplementary material for: Conservation and transmission of seed bacterial endophytes across generations following crossbreeding and repeated inbreeding of rice at different geographic locations
Source: Microbiologyopen. 2018 Jun 10;8(3):e00662. doi: 10.1002/mbo3.662 (PMC6436425; doi:10.1002/mbo3.662)
Supplement: Supplementary file 5 [file MBO3-8-e00662-s005.docx]

Table S1. Information on the rice (*Oryza* *sativa* ssp. indica) cultivars included in this study.

| Rice Cultivar | Parental Lines | Original source of the cultivar | Source of the seed samples | Special traits |
| --- | --- | --- | --- | --- |
| IR29 | IR833-6-2-1-1 I 11 (1561-149-1) / /1R1737 | Unknown | IRRI Philippines; RDA Korea | Salt-sensitive control |
| FL478 | IR29/POKKALI | Philippines | RDA Korea | Salt-tolerant control |
| Pokkali | Pure line | India | IRRI Philippines | Salt-tolerant gene source |
| (IR318) IR31868-64-2-3-3-3 | KHAO DAWK MALI 105/IR 9224-117-2-3-3//IR 9129-209-2-2-2-1 | IRRI Philippines | IRRI Philippines | Fertility restorer; yield enhancer |
| IC32  (IR 58443-6B-10-3) | AT 401/IR31868-64-2-3-3-3 | IRRI, International Rice Testing Program | RDA Korea | Salt-tolerant |
| AT401 | BG 94-1/POKKALI | Sri Lanka/India | IRRI Philippines; RDA Korea | Salt-tolerant |

Source: IRRI, Philippines; RDA, Korea.
