# Supplementary material for: Conservation and transmission of seed bacterial endophytes across generations following crossbreeding and repeated inbreeding of rice at different geographic locations
Source: Microbiologyopen. 2018 Jun 10;8(3):e00662. doi: 10.1002/mbo3.662 (PMC6436425; doi:10.1002/mbo3.662)
Supplement: Supplementary file 6 [file MBO3-8-e00662-s006.docx]

Table S2. Population of culturable seed bacterial endophytes of the parent (IR29 and Pokkali; IR31868 and AT401) and the RIL offspring (FL478 and IC32) rice cultivars belonging to *Oryza sativa* ssp. indica*.*

| Rice Cultivar | Population |
| --- | --- |
|  | Log CFU g^-1^ fresh weight |
| IR29 | 5.63±0.07 A |
| FL478 | 4.55±0.05 C |
| Pokkali | 5.49±0.05 B |
| IR31868-64-2-3-3-3 | 5.75±0.02 C |
| IC32 | 6.63±0.02 A |
| AT401 | 6.18±0.08 B |

Population is presented as means ± SE (standard error) from three replicates. Means with the same letter are not statistically significant at p<0.05 (Tukey’s test, SAS Version 9.4)..
