# Supplementary material for: Conservation and transmission of seed bacterial endophytes across generations following crossbreeding and repeated inbreeding of rice at different geographic locations
Source: Microbiologyopen. 2018 Jun 10;8(3):e00662. doi: 10.1002/mbo3.662 (PMC6436425; doi:10.1002/mbo3.662)
Supplement: Supplementary file 2 [file MBO3-8-e00662-s002.pdf]

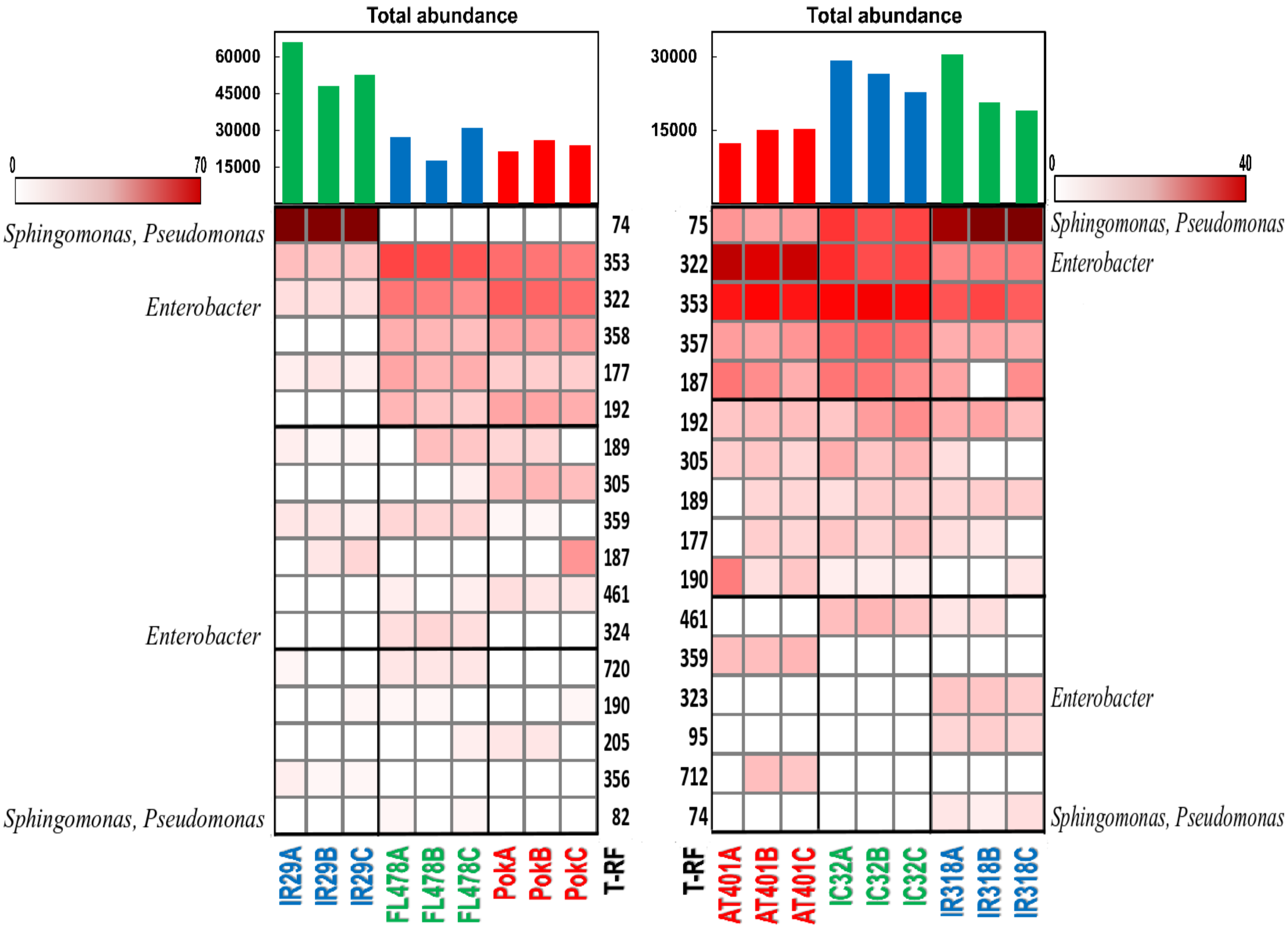

Total abundance
60000
45000
30000
15000
0
70
Sphingomonas, Pseudomonas
Enterobacter
Enterobacter
Sphingomonas, Pseudomonas
74
353
322
358
177
192
189
305
359
187
461
324
720
190
205
356
82
IR29A
IR29B
IR29C
FL478A
FL478B
FL478C
PokA
PokB
PokC
T-RF
Total abundance
30000
15000
0
40
75
322
353
357
187
192
305
189
177
190
461
359
323
95
712
74
Sphingomonas, Pseudomonas
Enterobacter
Enterobacter
Sphingomonas, Pseudomonas
T-RF
AT401A
AT401B
AT401C
IC32A
IC32B
IC32C
IR318A
IR318B
IR318C
